# Association of cooking fuel type with hypertension risk: a systematic review and meta-analysis

**DOI:** 10.1186/s12889-025-26168-5

**Published:** 2026-01-07

**Authors:** Jingna Li, Panke Su, Huashan Zhao, Lulu Chen, Xuejiao Li, Hongwei Jiang

**Affiliations:** 1 Department of Pharmacy, The First Affiliated Hospital, and College of Clinical Medicine of Henan University of Science and Technology, Luoyang, Henan 471000 China; 2 Quality Control Department, The First Affiliated Hospital, and College of Clinical Medicine of Henan University of Science and Technology, Luoyang, Henan 471000 China; 3 General Practice Department, The First Affiliated Hospital, and College of Clinical Medicine of Henan University of Science and Technology, Luoyang, Henan 471000 China; 4 Clinical Laboratory, The First Affiliated Hospital, and College of Clinical Medicine of Henan University of Science and Technology, Luoyang, Henan 471000 China; 5 Luoyang Key Laboratory of Clinical Multiomics and Translational Medicine,Henan Key Laboratory of Rare Diseases, Endocrinology and Metabolism Center, The First Affiliated Hospital, and College of Clinical Medicine of Henan University of Science and Technology, Luolong District, Guanlin Road, No. 636, Luoyang, 471003 China

**Keywords:** Hypertension, Cooking fuels, Meta-analysis

## Abstract

**Background:**

Hypertension is a major global health concern and a leading cause of mortality, contributing to functional and structural damage in vital organs. Emerging evidence suggests that household air pollution (HAP) from the combustion of cooking fuels, plays a critical role in influencing the prevalence and mortality rates of hypertension. This systematic review and meta-analysis aims to investigate the association between different cooking fuel types and hypertension risk.

**Methods:**

The study was conducted in accordance with the Preferred Reporting Items for Systematic Reviews and Meta-Analyses (PRISMA) guidelines and was registered with PROSPERO (CRD42024589847). A comprehensive literature search was performed across multiple databases, including PubMed, Embase, Cochrane, WOS, CNKI, VIP, Wanfang, and SinoMed up to September 3, 2024. Study quality was assessed using the Newcastle-Ottawa Scale (NOS) and the National Institutes of Health (NIH) Quality Assessment Tool. Statistical analyses were conducted using Stata 15.1 software. Heterogeneity was assessed using the Q-test and I² statistic, and an appropriate random-effects model (REM) or fixed-effects model (FEM) was applied based on the degree of heterogeneity observed.

**Results:**

The analysis revealed that the use of solid cooking fuels was significantly associated with increased odds of hypertension compared to clean cooking fuels (OR = 1.13, 95% CI: 1.03–1.25). Solid cooking fuel use was associated with higher systolic blood pressure (SMD = 0.12, 95% CI: 0.01–0.24), although the difference may be close to the critical level. However, the association with diastolic blood pressure was not significant (SMD = 0.05, 95% CI: -0.06–0.16). Neither subgroup analysis nor meta-regression identified sources of heterogeneity, while sensitivity analyses and subgroup analysis confirmed robust results.Sensitivity analyses confirmed the stability of the results, with no evidence of significant publication bias.

**Conclusions:**

This study provides evidence that solid cooking fuel use is associated with an elevated risk of hypertension. Despite limitations such as heterogeneity across study designs, the findings provide a strong scientific basis for policy interventions promoting a transition to cleaner cooking fuels. Future research should prioritize longitudinal or prospective study designs to enhance the quality of evidence and further elucidate this relationship.

**Supplementary Information:**

The online version contains supplementary material available at 10.1186/s12889-025-26168-5.

## Introduction

Hypertension is a major global health concern, affecting an estimated 1.2 billion individuals worldwide as of 2021 [1]. In 2019, it was implicated in 20% of global mortality [2, 3]. Delayed diagnosis of hypertension is associated with poor clinical outcomes [4]. According to global health data from the World Health Organization (WHO), only 21% of hypertensive patients achieve effective disease control [5]. A recent study from the Philippines projects that the economic burden of hypertension will rise from $1 billion in 2020 to $1.9 billion by 2050, underscoring its escalating socioeconomic impact [6].

Air pollution has emerged as a critical global health concern, with indoor air pollution contributing to approximately 3.8 million deaths annually worldwide [7]. Notably, the incidence and mortality associated with hypertension constitute a significant component of this disease burden [8–10]. Household cooking fuels can be broadly categorized into two groups: solid fuels (e.g., coal, wood, and crop residues) and clean fuels (e.g., natural gas and biogas). The combustion of solid fuels during routine household activities such as cooking, heating, and lighting generates household air pollution (HAP), which contains toxic emissions, including carbon monoxide and particulate matter (PM) [11, 12]. Exposure to these pollutants has been linked to a wide range of adverse health effects, including cognitive impairment, auditory dysfunction, depressive disorders, pulmonary diseases, atherosclerotic cardiovascular diseases, arthritis, lower urinary tract symptoms associated with benign prostatic hyperplasia, and detrimental effects on fetal and child growth and development [13–22].

The relationship between household air pollution and blood pressure remains a subject of ongoing debate. Empirical evidence suggests that a 10 µg/m³ increase in PM2.5 concentrations is associated with a 1.4 mmHg rise in systolic blood pressure (SBP), though no significant correlation has been observed for diastolic blood pressure (DBP) [23]. In Taipei, China, longitudinal studies have demonstrated a positive association between chronic exposure to PM and nitrogen oxides (NOx) and increased DBP among old residents [24]. However, some studies have reported no significant association between the use of solid cooking fuels and changes in blood pressure [25, 26]. In contrast, other investigations have indicated that adults using biomass fuels for cooking exhibit a heightened risk of developing hypertension [27, 28]. Conflicting findings also suggest that individuals relying on solid cooking fuels may have a lower likelihood of hypertension compared to those using cleaner energy sources [29, 30].

Given these inconsistencies, the present study employs a meta-analytical approach to systematically assess the association between cooking fuel type and hypertension risk, thereby providing a more comprehensive and rigorous scientific basis for understanding this complex relationship.

## Methods

This systematic review and meta-analysis was conducted in accordance with the Preferred Reporting Items for Systematic Reviews and Meta-Analyses (PRISMA) guidelines, a standardized framework designed to enhance methodological transparency and reporting completeness [31]. The study protocol was prospectively registered in the PROSPERO database (registration number: CRD42024589847; https://www.crd.york.ac.uk/PROSPERO/). This study follows the PRISMA statement, as detailed in Supplementary Table S1.

### Literature search strategy

A comprehensive literature search was conducted up to September 3, 2024, across major English-language databases (PubMed, Embase, Cochrane Library, and Web of Science) and Chinese-language databases (CNKI, VIP, Wanfang, and SinoMed). The search strategy incorporated a combination of controlled vocabulary (e.g., MeSH terms) and free-text keywords to identify studies examining the association between solid fuel use and hypertension incidence. The search terms included, but were not limited to, the following: Exposure-related terms: “cooking” OR “cookery” OR “clean household fuels” OR “solid fuel” OR “clean fuel” OR “cooking fuel” OR “biomass fuel” OR “cooking exposure”. Outcome-related terms: “vascular pressure” OR “systolic pressure” OR “systolic blood pressure” OR “systemic hypertension” OR “secondary hypertension” OR “salt hypertension” OR “salt high blood pressure” OR “pulse pressure” OR “blood pressure” OR “diastolic blood pressure” OR “hypertension” OR “diastolic pressure” OR “intravascular”. The comprehensive search strategy and methodology are detailed in Supplementary Tables S2–S9.

### Inclusion and exclusion criteria

Study selection was conducted in accordance with the PECOS (Population, Exposure, Comparison, Outcome, Study design) principle based on the following inclusion criteria: (1) Studies employing cohort, cross-sectional, or case-control designs; (2) Studies reporting odds ratios (ORs) with corresponding 95% confidence intervals (CIs) for the association between cooking fuel usage and hypertension, or providing sufficient raw data (number of hypertensive cases and total population in each group) to enable OR calculation; (3) Studies explicitly differentiating between solid fuels (e.g., wood, coal, biomass) and clean fuels (e.g., electricity, natural gas, biogas); (4) Observational studies conducted in general adult populations, without restrictions based on specific inclusion or exclusion criteria. Exclusion criteria were as follows: (1) Literature reviews or systematic analyses; (2) Case reports or editorial correspondence; (3) Studies involving non-adult populations; (4) Conference abstracts.

Hypertension was defined based on the presence of one or more of the following criteria: (1) SBP ≥ 140 mmHg and/or DBP ≥ 90 mmHg; (2) a confirmed hypertension diagnosis by a licensed healthcare provider; (3) current use of antihypertensive medications; or (4) a self-reported history of hypertension confirmed by a physician. Interventions: Participants in the intervention group used clean cooking fuels, including electricity, natural gas, or biogas, whereas those in the control group used solid fuels, such as wood, coal, or biomass.

### Literature screening process

All retrieved articles from the selected databases were consolidated, and duplicate records were systematically removed. Two independent reviewers (LXJ and ZHS) conducted a preliminary screening by evaluating titles and abstracts to exclude irrelevant studies. Articles meeting the preliminary screening criteria were retrieved for full-text review. Studies with inaccessible full texts were excluded. The remaining full-text articles were thoroughly assessed against the predefined inclusion criteria, and those meeting the inclusion criteria were included in the final analysis.

### Data extraction

Data extraction was independently conducted by two researchers (CLL and SPK) to ensure methodological rigor. Upon completion, a third reviewer (LJN) performed data reconciliation to verify consistency and completeness. Any discrepancies were resolved through a comprehensive review of the full text and discussion among all three researchers until a consensus was reached, ensuring the accuracy and reliability of the extracted data. Microsoft Excel was used to develop structured data extraction forms, systematically documenting all relevant information from the included studies. The extracted data encompassed the following variables: first author’s surname, publication year, study location (country), sample size, demographic characteristics (age and sex), hypertension diagnostic criteria, study design, grouping factors, exposure categories, subgroup analyses, risk estimates, and adjustments for confounding variables.

The Newcastle-Ottawa Scale (NOS) was employed to assess the quality and risk of bias in non-randomized studies, including cohort and case-control studies. This tool evaluates three key domains: selection of study groups, comparability between groups, and assessment of outcomes [32]. For cross-sectional studies, methodological quality was assessed using the standardized quality assessment tool developed by the National Heart, Lung, and Blood Institute of the National Institutes of Health (NIH). This structured, operationalized, evidence-based evaluation instrument consists of 10 specific criteria with response options of “yes”, “no”, and “unable to determine”. Based on the assessment results, the included studies were classified into three quality levels: low, medium, or high [33].

The application of these analytical tools enables a comprehensive and objective assessment of potential bias in research studies, thereby enhancing the accuracy and reliability of study quality evaluations in systematic reviews.

To standardize effect size measurements across studies, a systematic data analysis protocol was employed to convert hazard ratios (HRs) for hypertension risk into ORs. This conversion was conducted using a two-step approach: Step 1: Conversion of HR to relative risk (RR) using the following formula: [34]

$$\begin{aligned} \mathrm{RR} \approx \frac{1-0.5^{\sqrt{HR}}}{1-0.5^{\sqrt{1/HR}}} \end{aligned}$$

Step 2: Conversion of RR to OR using the following equation:[35]

$$\mathrm{OR}=\left(\left(1-\mathrm P\right)\ast\mathrm{RR}\right)/\left(1-\mathrm P\ast\mathrm{RR}\right)$$

where P denotes the prevalence rate of hypertension within the population subgroup utilizing solid cooking fuels.

### Statistical analysis

All statistical analyses were conducted using Stata software (version 15.1; Stata Corp, College Station, TX, USA). Heterogeneity among studies was assessed using both the Q-test and the I² statistic, with I² quantifying the proportion of total variability attributable to between-study heterogeneity. A random-effects model (REM) was applied when the I² > 50% and *p* < 0.05; otherwise, a fixed-effects model (FEM) was employed. For continuous outcomes, effect sizes were reported as Standardized Mean Differences (SMDs) and visualized using forest plots, while dichotomous outcomes were analyzed using ORs. All effect estimates were reported with 95% confidence intervals (CIs). In instances of significant heterogeneity (I² > 50% and *p* < 0.05 for the Q-test), sensitivity analyses were conducted to assess the robustness of the results. Publication bias was evaluated using funnel plots and Egger’s test when the number of included studies exceeded 10. If publication bias was detected, the trim-and-fill method was applied to adjust the effect estimates accordingly.

To systematically evaluate potential differences in effect sizes across subgroups, we calculated and pooled effect sizes along with within-group heterogeneity (I²) for each subgroup, presenting these results in tabular format. Subsequently, subgroup differences were assessed using the test for subgroup differences. In addition, meta-regression analysis was conducted based on study type (cross-sectional, case-control, and cohort studies) and different quality levels of studies to further explore the sources of heterogeneity. A between-group p-value ≤ 0.05 was considered indicative of statistically significant differences between subgroups.

## Results

### Included studies

**Fig. 1:**
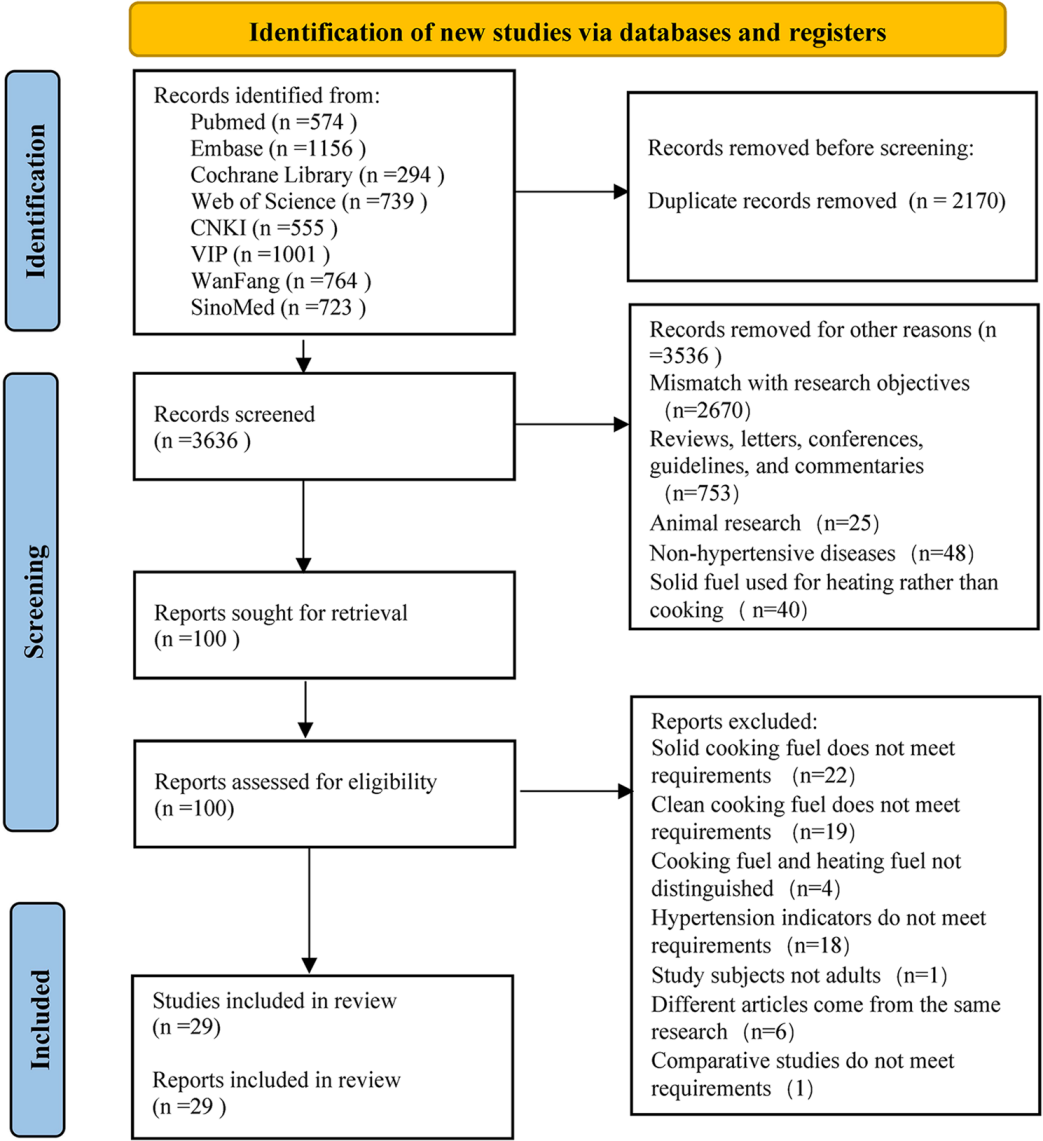
Flow chart of study selection

A total of 5,806 articles were initially identified through the literature search. After the removal of 2,170 duplicates, 3,636 unique records were screened. Following a review of the titles and abstracts, 3,536 studies were excluded as irrelevant. Full-text articles for the remaining 100 studies were retrieved and thoroughly reviewed. After the full-text evaluation, 71 were excluded based on predefined exclusion criteria: 22 studies used only solid cooking fuels; 19 studies employed unclean cooking fuels; 4 studies failed to differentiate between cooking and heating fuels; 1 study focused on non-adult populations; 6 studies had duplicate data sources; and 19 studies had non-comparative study designs. Consequently, 29 articles met the inclusion criteria and were included in the final analysis [26, 28–30, 36–60](Fig. 1).

### Description of basic information

This meta-analysis included 29 peer-reviewed studies published between 2010 and 2023, comprising a total sample size of over 342,000 participants. The sample sizes of individual studies ranged from 123 to 97,942 participants.

The included studies consisted of 18 cross-sectional studies, along with 11 longitudinal cohort and case-control studies. Among them, 14 studies focused exclusively on female populations, while the remaining 15 studies included both male and female participants. Geographically, the studies spanned multiple regions, including Asia, Africa, Latin America, Eastern Europe, and Southeast Asia, with a particular concentration in developing countries. Notably, there were no studies exclusively from developed countries in Europe or North America. While the majority of studies were conducted in Asian countries, African countries demonstrated a broader geographic distribution, although they were represented by fewer single-country studies, suggesting wide spatial coverage but limited depth of regional analysis. China and India were notably the most frequently represented countries, as detailed in Table 1. The studies investigated the use of traditional solid fuels, including coal, wood, kerosene, charcoal, and biomass, and compared them with cleaner energy alternatives such as electricity, natural gas, biogas, liquefied petroleum gas, and solar power.

**Table 1:** Basic information

General Information			Demographic information of population				Research characteristics		Outcome
First Author	Publication Year	Country	Sample Size	Age (Mean ± SD or Range)	Gender(M/F)	Criterion	Type study	Follow up(Month)	
Khan	2021	Bangladesh	6543	18–34(47.6*) 35–49(29.5*) 50–64(15.8*) ≥ 65 (7.1*)	100%femal	140/90	Cross-sectional study	24	①②
Zhang	2023	China	1453	77.6 ± 8.8	50.7%/49.3%	140/90	Cohort study	84	①②
Abba	2022	Albania	20,846	15–24(4511 ) 25–34(4082) 35–44(4091) 45–54(5257) 55–64(2905)	5988/14,858	140/90	Cross-sectional study	/	①
Alexander	2017	Nigerian	324	Intervention group 28.0 ± 6.1 Control group 27.9 ± 5.4	100*femal	140/90	Case-control study	28	①②③
Arku	2020	India, Saudi Arabia	43,313	50.6 ± 9.8	42%/58%	140/90	Cross-sectional study	168	①②④
Ayeben	2023	Benin, Burundi, Cameroon, Ghana, etc.	97,942	15–49	100%femal	Questionnaire survey	Cross-sectional study	96	①
Bellows	2020	China	22,118	18–80	100%femal	/	Cohort study	132	②
Dutta	2011	India	480	22–41	100%femal	140/90	Cross-sectional study	72	①②③⑥
Islam	2023	Bangladesh	2182	35.87 ± 11.09	100%femal	140/90	Cross-sectional study	12	①②
Kanagasabai	2022	China	753	40–79	337/416	140/90	Cohort study	14	①②
Lin	2021	China	10,450	clean fuel 58.55 ± 9.52 solid fuel 59.92 ± 9.35	4957/5493	140/90, doctor diagnosed, taking antihypertensive medication	Cross-sectional study	10	①②
Liu	2022	China	44,862	51.1 ± 9.65	22,086/22,776	140/90, doctor diagnosed, taking antihypertensive medication	Cross-sectional study	60	①②
Mitra	2023	India	123	18–68	100%femal	140/90, doctor diagnosed	Case-control study	8	①②
Neupane	2015	Nepal	519	30–80	100%femal	140/90	Cross-sectional study	120	①②
Ofori	2018	Nigerian	389	38.7 ± 14.1	100%femal	140/90	Cross-sectional study	/	①②
Peng	2022	China	10,400	44.44 ± 15.42	4531/5869	140/90, doctor diagnosed, taking antihypertensive medication	Cohort study	/	①②
Painschab	2013	Peru	266	≥ 35	123/143	/	Cross-sectional study	8	①②
Su	2022	China	5600	42.00 ± 14.18	2556/3044	140/90, doctor-diagnosed, taking antihypertensive medication, self-reported	Cohort study	60	①
Yang	2023	China	15,616	≥ 18	100%femal	140/90, doctor diagnosed, taking antihypertensive medication	Cross-sectional study	36	①
He	2023	China	38,839	50.1 ± 9.5	16,309/22,530	140/90, doctor-diagnosed, self-reported	Cross-sectional study	60	①②
Zhong	2022	China	1248	46.42 ± 15.07	550/698	140/90, doctor diagnosed, taking antihypertensive medication	Cohort study	54	①②
Younger	2023	Guatemala, Peru, India, Rwanda	3195	25.4 ± 4.5	100%femal	140/90	Case-control study	23	⑦
Yu	2022	China	8067	>65	3610/4457	140/90	Cohort study	12	①②
Wylie	2015	India	1369	/	100%femal	139/89	Cross-sectional study	12	①②
Weber	2020	Ghana	819	28.3 ± 5	100%femal	Preeclampsia, HELLP syndrome	Cohort study	24	⑦
Tiwana	2020	Nepal	299	40–70	100%femal	140/90, doctor diagnosed	Cross-sectional study	24	①②
Tawiah	2022	Ghana	350	18–72	341/9	140/90	Cross-sectional study	3	①②
Juntarawijit	2020	Thailand	1078	53.04 ± 12.93	170/908	self-reported	Cross-sectional study	1	①
Gu	2014	China	2669	48.4 ± 15.7	100%femal	140/90	Cross-sectional study	/	①

①Hypertension, ②SBP, DBP; ③Prehypertension; ④Mean Arterial Pressure; ⑤Pulse pressure; ⑥Normal blood pressure; ⑦Preeclampsia

The quality assessment of cohort and case-control studies revealed that 7 studies were categorized as high quality, 2 as moderate quality, and 2 as low quality, yielding an average quality score of 7.5 based on the Newcastle-Ottawa Scale (NOS). Additionally, one study was classified as low quality according to the National Institutes of Health (NIH) assessment criteria. Detailed results are presented in Supplementary Tables S10 - S12.

### Association between cooking fuel types and hypertension risk

**Fig. 2:**
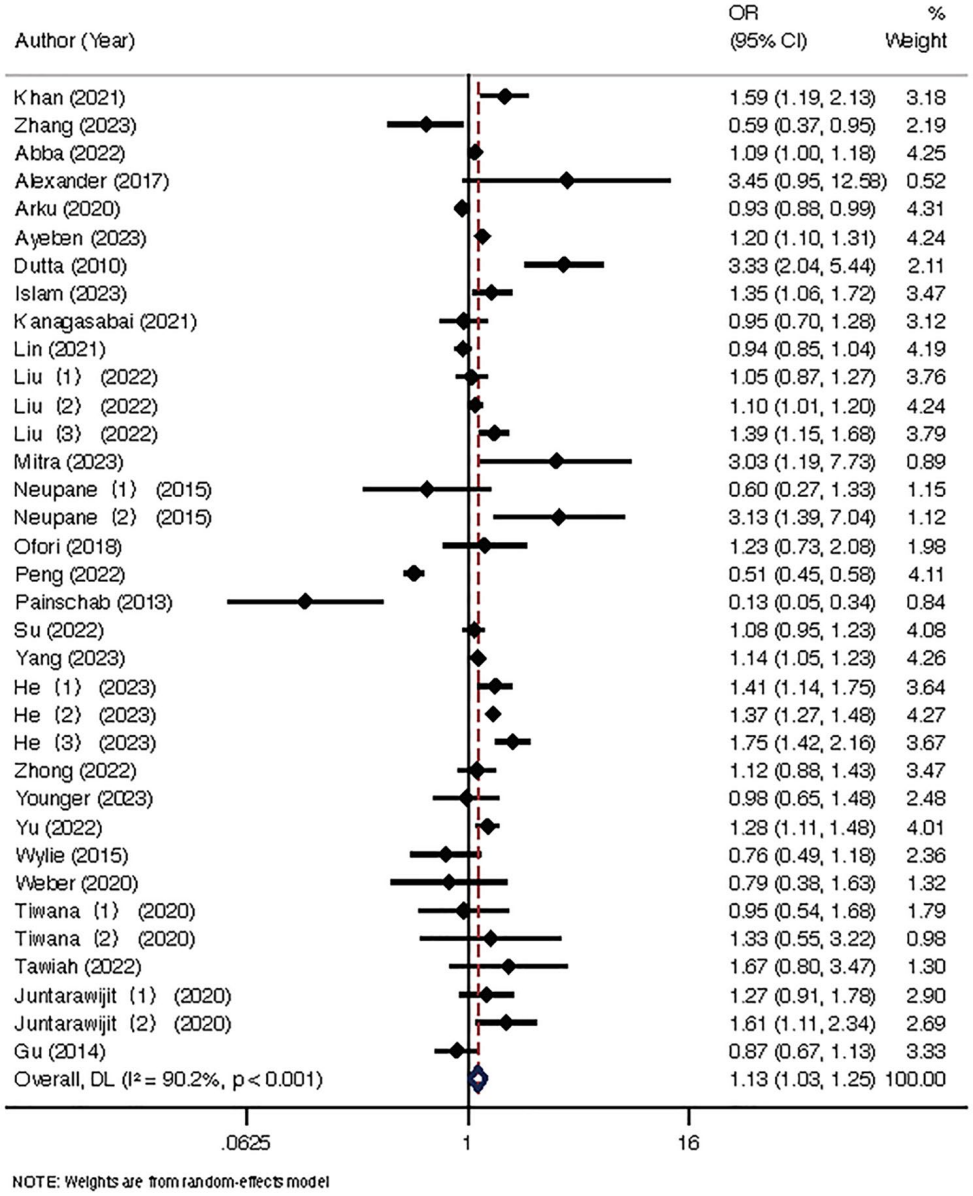
Forest plot of the effect of solid cooking fuels on hypertension risk. Note: Liu (1) represents the risk estimate of solid fuel vs. liquefied gas; Liu (2) represents the risk estimate of solid fuel vs. natural gas; Liu (3) represents the risk estimate of solid fuel vs. Electricity; Neupane (1) represents the risk estimate of solid fuel vs. biogas for 30-50 years; Neupane (2) represents the risk estimate of solid fuel vs. biogas >50 years; He (1) represents the risk estimate of solid fuel vs. liquefied gas by age; He (2) represents the risk estimate of solid fuel vs. natural gas; He (3) represents the risk estimate of solid fuel vs. Electricity; Tiwana (1) represents the risk estimate of solid fuel vs. liquefied gas; Tiwana (2) represents the risk estimate of solid fuel vs. Biogas; Juntarawijit (1) represents the risk estimate of solid fuel vs. liquefied gas users; Juntarawijit (2) represents the risk estimate of solid fuel vs. liquefied gas users’ families

The results of the meta-analysis revealed a statistically significant association between the use of solid cooking fuels and increased odds of hypertension when compared to the use of clean cooking fuels (OR = 1.13, 95% CI: 1.03–1.25), as illustrated in Fig. 2.

### Influence of cooking fuel types on blood pressure readings

**Fig. 3:**
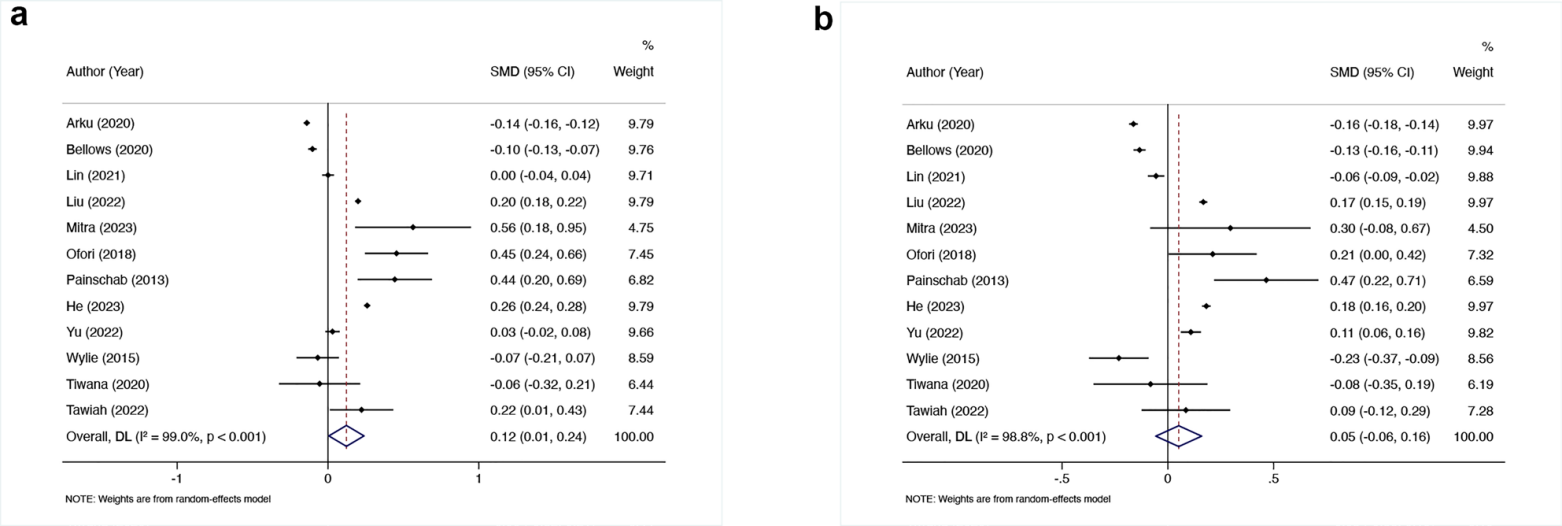
**a** Forest plot of the effect of solid cooking fuels on systolic blood pressure **b** Forest plot of the effect of solid cooking fuels on diastolic blood pressure

A meta-analysis of 13 studies demonstrated no statistically significant differences in blood pressure parameters, including SBP and DBP, between individuals using solid cooking fuels and those utilizing cleaner alternatives. Specifically, the analysis revealed an extremely small difference in SBP between the two groups (SMD = 0.12, 95% CI: 0.01–0.24, I² = 99%), as illustrated in Fig. 3a. Similarly, for DBP, no significant difference was observed between solid cooking fuel users and those employing cleaner alternatives (SMD = 0.05, 95% CI: -0.06-0.16, I² = 98.8%), as illustrated in Fig. 3b.

### Subgroup analysis and meta-regression analysis

Subgroup analysis was performed to explore potential sources of heterogeneity across studies, as different study types may yield varying results. The present study identified a substantial representation of Chinese research (40%), prompting stratified subgroup analyses by country (China vs. other countries) to examine possible regional and developmental differences. Given the cumulative nature of cooking fuel exposure on blood pressure, stratification by follow-up duration was conducted to assess the progressive effects of indoor air pollution over time. Gender-based subgroup analysis was performed to explore potential sex-specific health outcomes, and due to variability in the definitions of hypertension, a subgroup analysis by blood pressure measurement criteria was undertaken to assess its impact on study outcomes. To address heterogeneity across study designs, subgroup analysis by study type was also conducted to enhance result interpretability. Furthermore, subgroup analysis by hypertension classification criteria was implemented to examine the influence of different hypertension recording methods. Notably, certain subgroups exhibited divergent outcomes, particularly in the analysis of SBP when stratified by study type and follow-up duration, and DBP when stratified by gender. These differences were likely attributable to unequal sample size distributions across subgroups. Importantly, subgroup analyses by study country, study design, follow-up duration, hypertension diagnostic criteria, and participant gender did not significantly alter the primary study findings. Comprehensive dataset details are presented in Table 2.

**Table 2:** Subgroup analysis results

Subgroup		SBP			DBP			OR		
		Number of studies	SMD,95%CI	I^2^	Number of studies	SMD,95%CI	I^2^	Number of studies	OR,95%CI	I^2^
Country	China	5	0.08(-0.06,0.22)	99.20%	5	0.05(-0.07,0.18)	99.10%	15	1.07(0.92,1.24)	94.20%
	Other countries	7	0.18(-0.03,0.39)	92.10%	7	0.06(-0.12,0.23)	87.70%	20	1.22(1.06,1.40)	81.90%
	Heterogeneity between group	*p* = 0.433			*p* = 0.982			*p* = 0.219		
Type_study (Ts)	Cross-sectional studies	9	0.14(-0.00,0.28)	99.10%	9	0.05(-0.07,0.18)	99%	24	1.21(1.09,1.34)	86.80%
	Cohort studies	2	-0.04(-0.17,0.09)	95.50%	2	-0.01(-0.25,0.22)	98.70%	7	0.87(0.62,1.23)	95%
	Case-control studies	1	0.56(0.18,0.95)	0	1	0.3(-0.08,0.67)	0	4	1.32(0.89,1.97)	61.70%
	Heterogeneity between group	*p* = 0.007			*p* = 0.395			*p* = 0.175		
FUT	≥ 3 years	4	0.05(-0.15,0.26)	99.70%	4	0.01(-0.17,0.20)	99.60%	14	1.23(1.08,1.39)	90%
	≤ 3 years	7	0.08(-0.01,0.17)	76.50%	7	0.05(-0.07,0.17)	89.10%	17	1.15(1.00,1.32)	73.40%
	Unclear	1	0.45(0.24,0.66)	0	1	0.21(0,0.42)	0	4	0.86(0.53,1.40)	97.10%
	Heterogeneity between group	*p* = 0.004			*p* = 0.321			*p* = 0.329		
Gender	female	5	0.12(-0.01,0.34)	89.50%	5	-0.04(-0.19,0.11)	77.10%	16	1.24(1.07,1.43)	69.70%
	male and female	7	0.13(-0.02,0.28)	99.30%	7	0.1(-0.04,0.23)	99.20%	19	1.08(0.94,1.23)	93.70%
	Heterogeneity between group	*p* = 0.952			*p* = 0.201			*p* = 0.159		
Criteria	140/90	5	0.07(-0.07,0.21)	95.00%	5	-0.01(-0.18,0.16)	96.70%	16	1.15(1.00,1.33)	81.50%
	Diagnosis by doctors and/or take any hypertensive medications	4	0.13(-0.04,0.29)	96.60%	4	0.06(-0.12,0.24)	97.20%	11	1.04(0.87,1.26)	93.50%
	Self-report	3	0.18(-0.12,0.48)	99.50%	3	0.15(-0.12,0.41)	99.40%	8	1.29(1.09,1.52)	81.50%
	Heterogeneity between group	*p* = 0.741			*p* = 0.622			*p* = 0.239		

Furthermore, meta-regression analysis was conducted based on different study types (cross-sectional, case-control, and cohort studies) and their corresponding low, medium, and high quality levels. The results showed that neither the study type nor the quality level significantly affected the conclusions of this study (*P* > 0.05).

### Analysis of sensitivity and publication bias

The sensitivity analysis for hypertension risk demonstrated consistent robustness, as evidenced by the results shown in Supplementary Figure S1. Excluding any individual study did not significantly alter the overall hypertension risk estimates. The Egger test for publication bias yielded a p-value of 0.469, suggesting no substantial evidence of publication bias. Furthermore, the funnel plot analysis for hypertension risk, depicted in Supplementary Figure S2, confirmed the absence of significant publication bias.

Similarly, sensitivity analyses for blood pressure parameters (SBP and DBP) exhibited robust findings. The results for SBP and DBP are detailed in Supplementary Figures S3 and S4, respectively. The exclusion of any single study did not significantly affect the outcomes for either SBP or DBP. The Egger test p-values for SBP and DBP were 0.974 and 0.895, respectively, both exceeding the threshold of 0.05, reinforcing the absence of significant publication bias.

## Discussion

Previous systematic reviews and meta-analyses have preliminarily explored the association between solid fuel use and the risk of hypertension [13, 61, 62] (Table S13), but these studies were mostly limited to specific populations (e.g., including only women) or were published before 2020. Between 2020 and 2024, more large-sample studies have been published, providing new data for elucidating the relationship between different fuel types and hypertension.

It is noteworthy that while early high-quality studies have found a significant association between solid fuel heating and elevated blood pressure, no clear association is observed between exposure to solid cooking fuel and hypertension [26], suggesting that the biological effects of exposure to cooking fuel may have unique pathways. Therefore, this study conducted a systematic meta-analysis of 29 studies (involving 324,000 participants) and found that the use of solid cooking fuel significantly increased the risk of hypertension (OR = 1.13). However, no significant differences were observed between groups in continuous measurements of systolic and DBP.

This seemingly contradictory result can be explained from the perspective of biological mechanisms. The use of solid fuels is predominantly associated with the emission of hazardous pollutants, including polycyclic aromatic hydrocarbons (PAHs), particulate matter (PM), nitrous oxide (NOx), carbon monoxide (CO), and sulfur dioxide (SO2). Comparative analyses indicate that transitioning to cleaner energy alternatives, such as liquefied petroleum gas (LPG), biogas, or electricity, significantly reduces daily exposure to PM and aromatic compounds, in contrast to the ongoing use of traditional solid fuels [63–65].Pollutants such as PAHs and PM are released from the combustion of solid fuels. These pollutants induce oxidative stress and consume nitric oxide (NO) with a vasodilatory effect, which is then converted into cytotoxic peroxynitrite, thereby causing endothelial dysfunction and arteriosclerosis. This process is the key pathological basis for the development of hypertension [66–69]. Because cooking is usually intermittent and short-term, the resulting blood pressure changes may be fluctuating and cumulative. In the early stages, it is more likely to manifest as functional dysregulation rather than a sustained increase in blood pressure, making it difficult to capture in cross-sectional measurements. However, in the long term, it can eventually lead to an increased risk of developing hypertension [70].

Therefore, this study shows that although the use of solid cooking fuels did not show a difference in immediate blood pressure measurements, it still significantly increases the risk of hypertension over the long term through the aforementioned biological pathways. This finding strengthens the public health evidence for the shift from solid cooking fuels to clean energy and also points the future research directions for intervention targeting early changes in vascular function.

Our comprehensive meta-analysis of previous observational studies revealed a significant positive correlation between solid fuel use and hypertension incidence compared to cleaner fuel alternatives, though the effects on SBP and DBP measurements were minimal. The analysis demonstrated that solid cooking fuel use was associated with 13% increased odds of hypertension (OR = 1.13, 95% CI: 1.03–1.25). Given the widespread use of solid cooking fuels in certain populations, even this modest 13% increase in risk could contribute to a substantial burden of hypertension cases at the population level.

The absence of a significant association between cooking fuel type and blood pressure measurements in this study may be attributed to several methodological factors. Firstly, the relatively small sample size for blood pressure data, with only 12 out of the 29 studies providing relevant measurements, may have limited the statistical power to detect meaningful associations between solid fuel use and blood pressure outcomes despite a potential relationship with hypertension risk. Secondly, the considerable heterogeneity in follow-up durations across studies could have hindered the assessment of long-term cardiovascular effects. Shorter observational periods may have led to the selective manifestation of hypertension among high-risk individuals within the study duration, potentially biasing the comprehensive evaluation of the impact of cooking fuel type on blood pressure parameters.

Sensitivity and subgroup analyses consistently yielded results that did not significantly alter the primary conclusions of this study. This suggests that, despite heterogeneity in study design, sample size, and geographic distribution, these factors did not compromise the robustness of the core findings. Specifically, the analysis consistently demonstrated a statistically significant increase in hypertension risk among users of solid cooking fuels, regardless of stratification by gender, age group, or specific fuel types. The methodological consistency across these analyses strengthens the reliability of the findings, providing robust evidence to support public health policy initiatives aimed at transitioning from solid fuels to cleaner energy alternatives.

The current study acknowledges several methodological limitations that warrant consideration. First, the majority of the included studies employed case-control or cross-sectional designs, which, while valuable for identifying potential associations, are inherently limited in their ability to establish causal relationships when compared to longitudinal or prospective cohort studies. The latter study designs offer a greater capacity for controlling confounding variables and tracking long-term health outcomes in individuals exposed to specific cooking fuels. Therefore, future research should prioritize the use of longitudinal or prospective cohort studies to strengthen the evidence base. Furthermore, the geographical distribution of the included studies was predominantly focused on Asian and African regions, which may limit the generalizability of the findings. Future investigations should incorporate epidemiological data from a broader range of geographical regions, including Europe, the Americas, and Oceania. Such an approach would enable a more comprehensive evaluation of the geographical heterogeneity in the association between cooking fuel exposure and hypertension, providing a stronger evidence base for global public health interventions.

The studies included in this meta-analysis exhibited considerable heterogeneity, potentially due to variations in demographic characteristics (such as age distribution and gender composition), geographical settings (urban vs. rural populations), diverse cooking practices, and methodological inconsistencies in exposure assessment. To mitigate the impact of inter-study heterogeneity, we employed subgroup analysis and stringent inclusion criteria. However, heterogeneity was not completely eliminated. Through subgroup analysis and meta-regression on factors such as fuel type, study design, and the quality of the original study, we systematically explored potential sources of heterogeneity. Nonetheless, no sources of heterogeneity were found. Nevertheless, the results of subgroup analysis and sensitivity analysis consistently indicated that the core findings of this study remained significantly unchanged. This result suggests that despite some heterogeneity, the significant association between the use of solid fuels and increased risk of hypertension remains robust.

It is important to note that high heterogeneity is a common phenomenon in meta-analyses, especially when including studies with different research designs, populations, and methodologies. Statistically, heterogeneity simply reflects inconsistencies in the findings of individual studies, which is particularly prevalent in observational research. As shown in the Cochrane Handbook (Sect. 12.1) [71], even with high heterogeneity, meta-analysis can still provide important scientific insights if properly interpreted. Therefore, while we were unable to fully identify the specific sources of heterogeneity, the main findings of this study are consistent in subgroup analyses, providing significant references.

Regarding reverse causality, it is still possible that pre-existing hypertension might lead individuals to continue using solid cooking fuels (e.g., decreased earning capacity due to illness). Nevertheless, it may not be the primary factor due to the following reasons: (1) the biological pathways by which solid fuel pollutants cause inflammation and endothelial dysfunction are well-defined and precede hypertension; and (2) some studies have shown a dose-response relationship between exposure and risk, supporting that exposure to cooking fuels precedes hypertension. Nonetheless, cross-sectional data alone are insufficient to fully clarify this issue. Therefore, further investigation into the relationship between solid cooking fuels and the risk of hypertension is needed.

Therefore, this study reveals a robust association between the use of solid cooking fuels and hypertension, rather than a definitive causal relationship. Future research, including prospective cohort studies and randomized intervention trials, is needed to definitively establish their causal association and to more precisely quantify the confounding effects of other social and environmental factors.

In conclusion, our findings clearly indicate that air pollution from household solid fuels is a key factor contributing to the prevalence of hypertension, and urgent policy responses are required.

A strategic policy package is needed to translate this finding into effective preventative measures. It is recommended to integrate clean fuel subsidies into routine public health initiatives. Fuel conversion should be combined with health education to jointly reduce risky health behaviors. Longitudinal studies should be conducted to trace the early-life origins of hypertension. The impact of all future intervention policies, such as interruption time-series analyses, should be rigorously evaluated. Such collaborative efforts are key to primary prevention, shifting the focus from treating hypertension to preventing its onset.

## Supplementary Information

Supplementary Material 1: Figure S1 Sensitivity analysis for the risk of hypertension. Figure S2 Funnel plot for OR. Figure S3 Sensitivity analysis for SBP. Figure S4 Sensitivity analysis for DBP.

Supplementary Material 2: Table S1 PRISM checklist. Table S2 Pubmed search strategy. Table S3 Embase search strategy. Table S4 Cochrane Library search strategy. Table S5 Web of Science search strategy. Table S6 CNKI search strategy. Table S7 VIP search strategy. Table S8 Wanfang search strategy. Table S9 SinoMed search strategy. Table S10 Quality assessment of case-control studies. Table S11 Quality assessment of cohort studies. Table S12 Quality assessment of cross-sectional studies. Table S13 Previous meta-analysis summary table.

## Data Availability

All data generated or analyzed during this study are included in this published article and its supplementary information files.

## Abbreviations

HAP: Household Air Pollution
PRISMA: Preferred Reporting Items for Systematic Reviews and Meta-Analyses
OR: Odds Ratio
HR: Hazard Ratio
CI: Confidence Interval
NOS: Newcastle-Ottawa Scale
NIH: National Institutes of Health
REM: Random-effects Model
FEM: Fixed-effects Model
PM: Particulate Matter
VOCs: Volatile Organic Compounds
Nox: Nitrogen Oxides
SBP: Systolic Blood Pressure
DBP: Diastolic Blood Pressure
SMD: Standardized Mean Difference
PAHs: Polycyclic Aromatic Hydrocarbons
LPG: Liquefied Petroleum Gas
ROS: Reactive Oxygen Species
NO: Nitric Oxide
H_2_O_2_: Hydrogen Peroxide
