# Supplementary material for: Association of cooking fuel type with hypertension risk: a systematic review and meta-analysis
Source: BMC Public Health. 2026 Jan 7;26:386. doi: 10.1186/s12889-025-26168-5 (PMC12853769; doi:10.1186/s12889-025-26168-5)
Supplement: Supplementary file 1 — Supplementary Material 1: Figure S1 Sensitivity analysis for the risk of hypertension. Figure S2 Funnel plot for OR. Figure S3 Sensitivity analysis for SBP. Figure S4 Sensitivity analysis for DBP. [file 12889_2025_26168_MOESM1_ESM.docx]

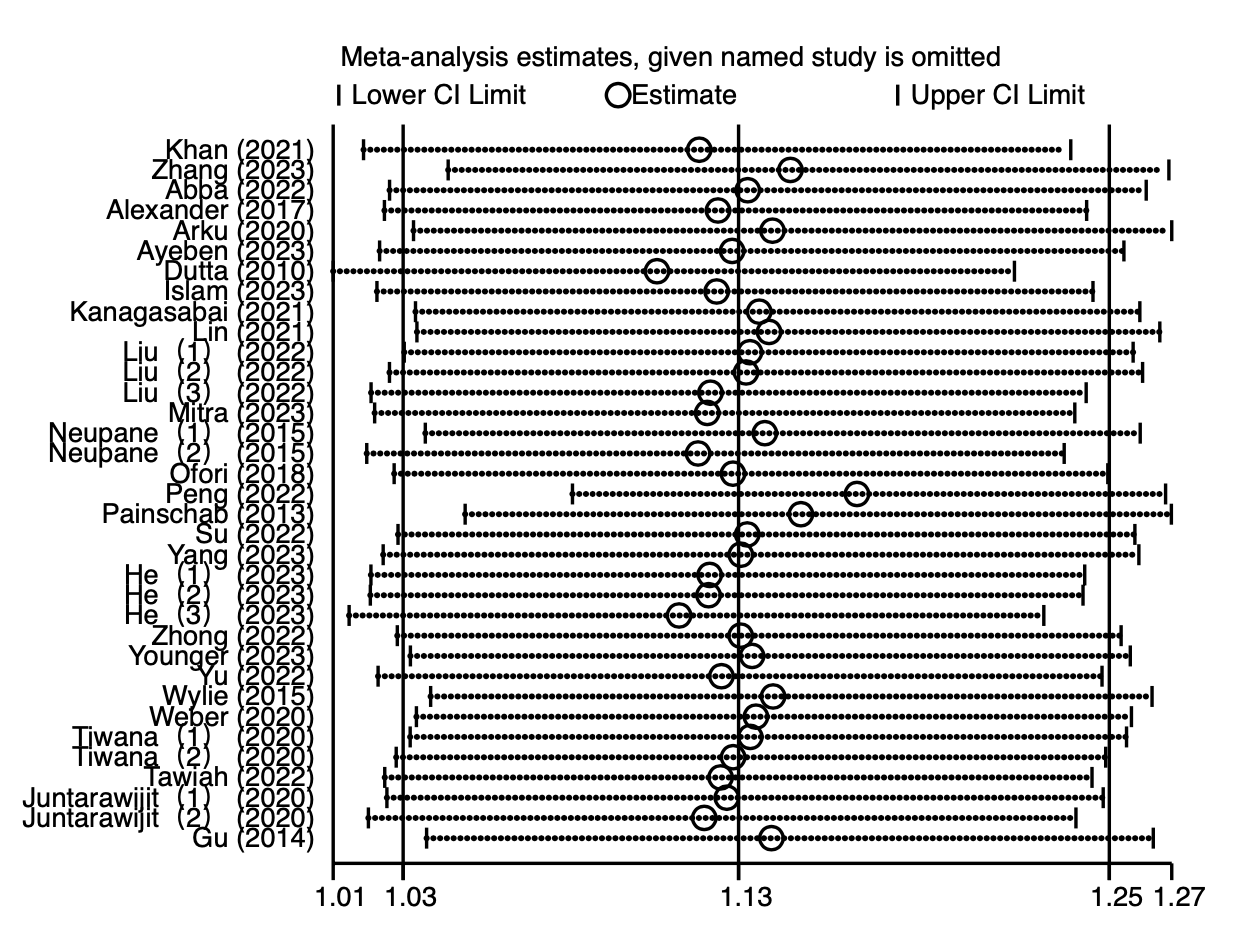


Figure S1 Sensitivity analysis for the risk of hypertension


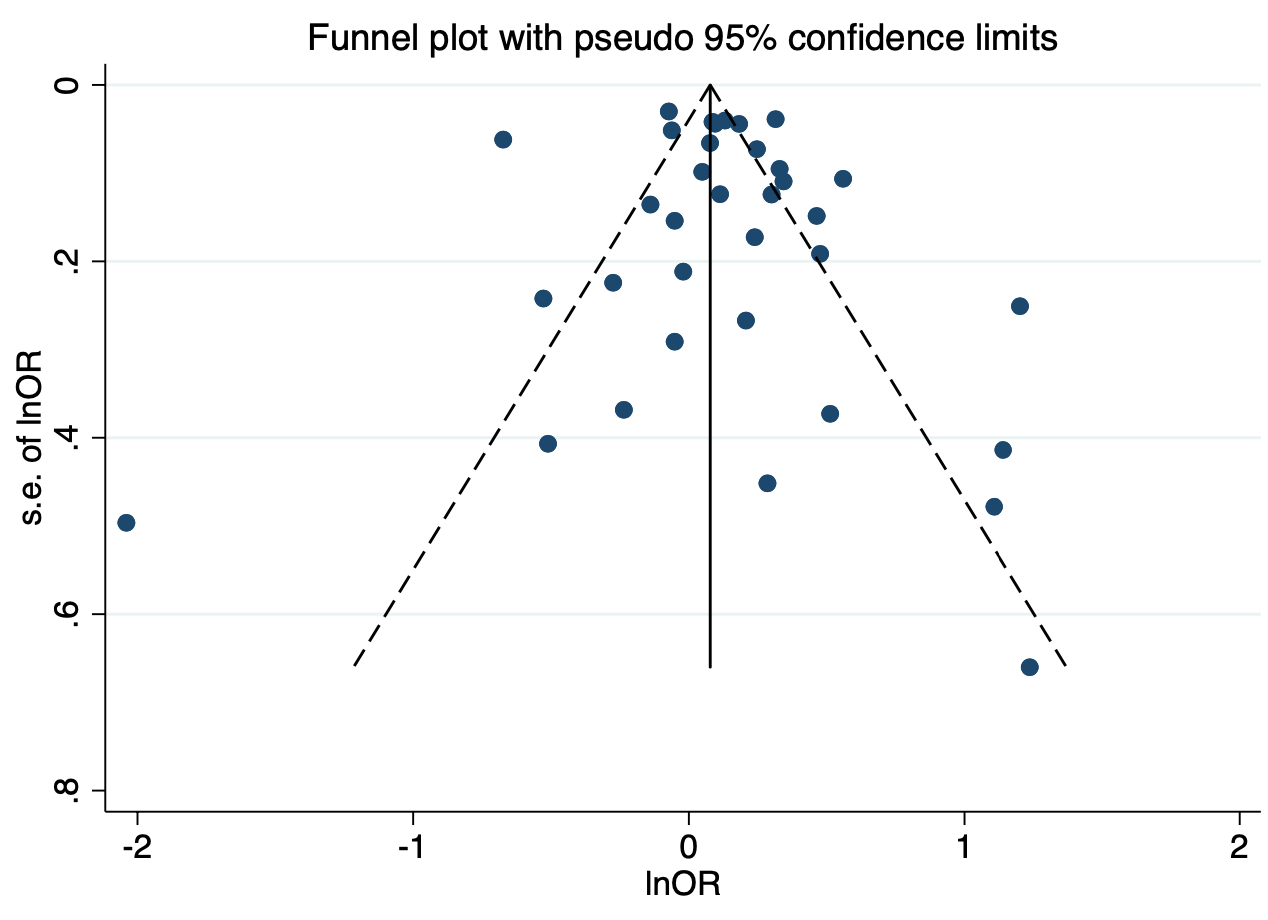


Figure S2 Funnel plot for OR


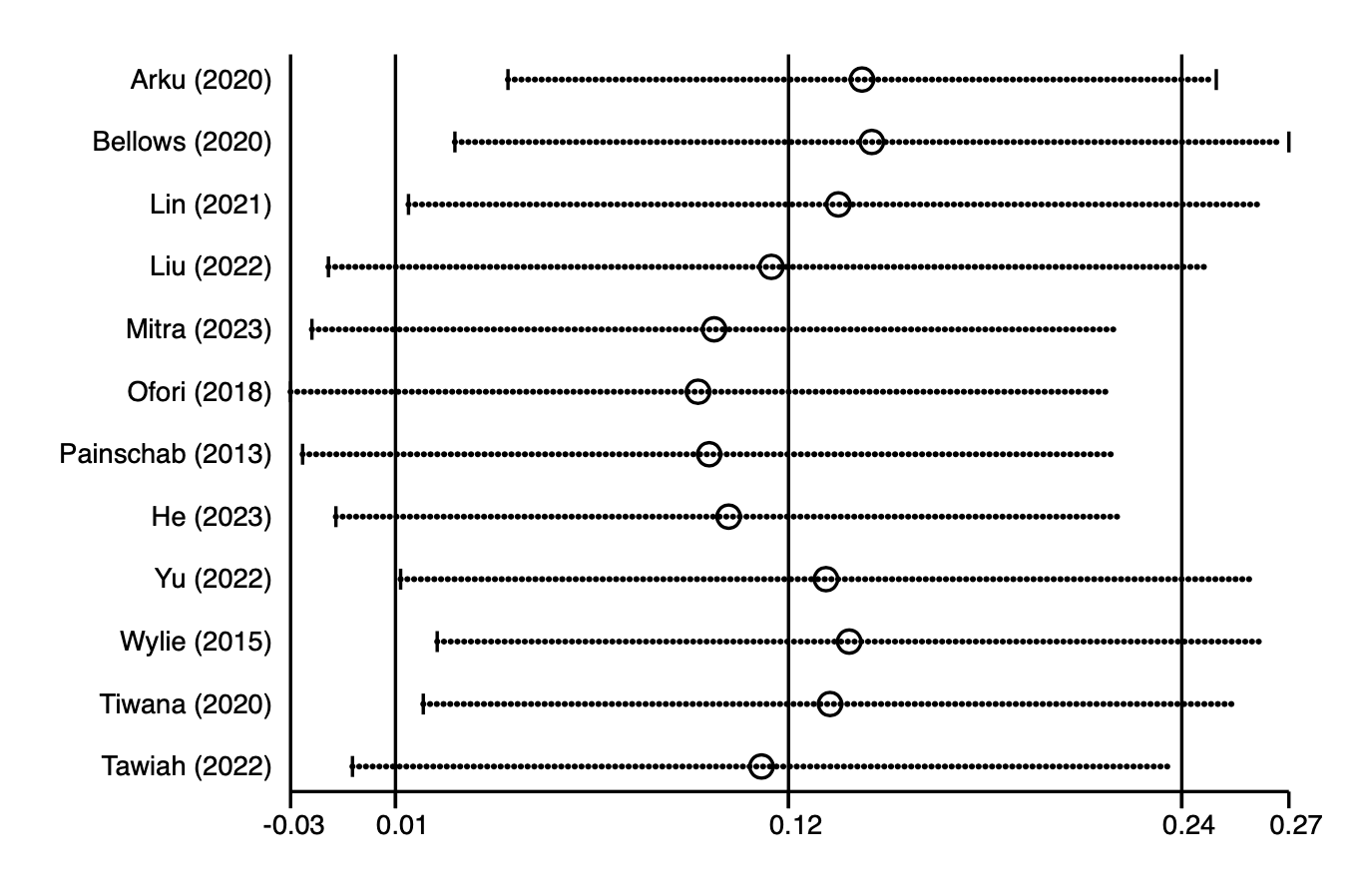


Figure S3 Sensitivity analysis for SBP


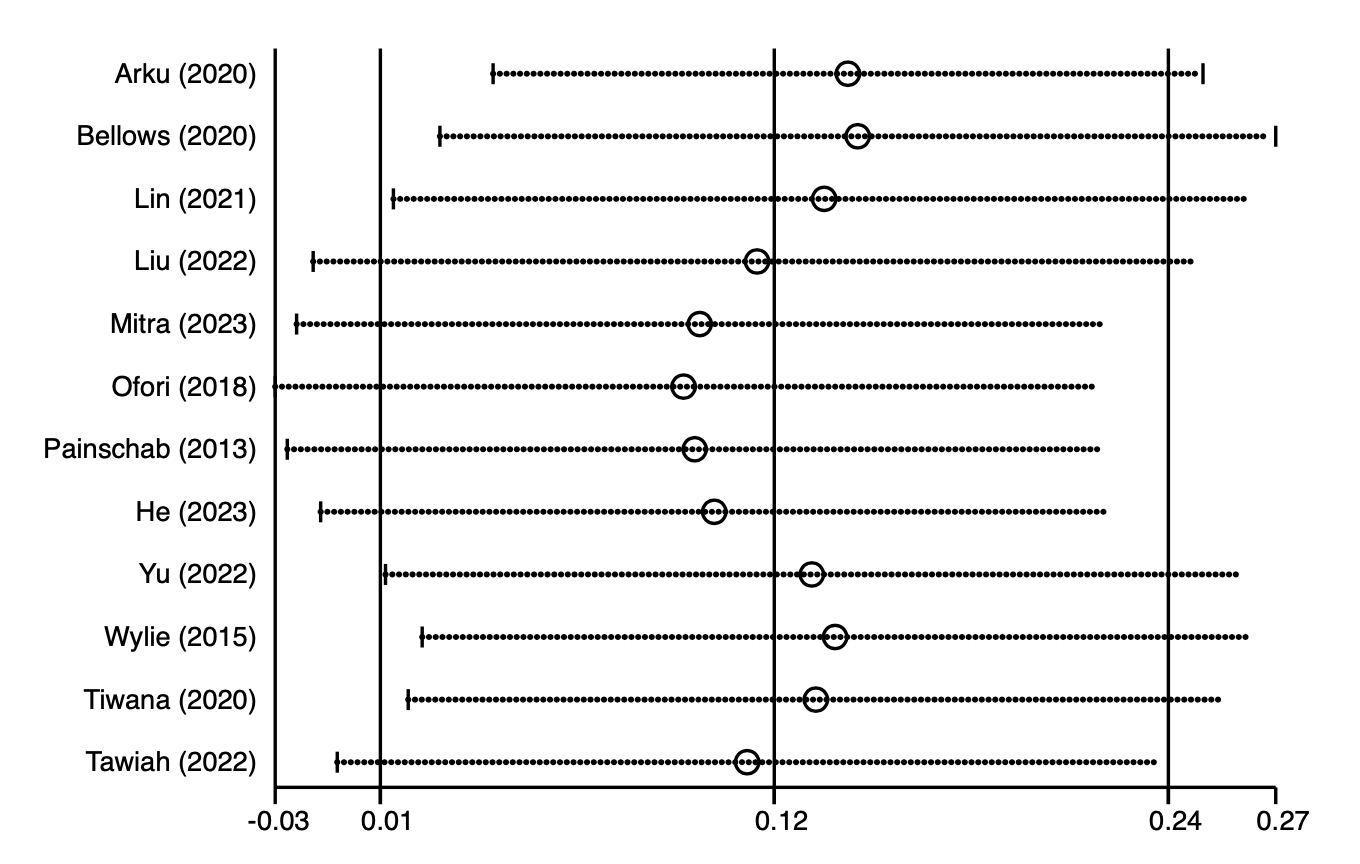


Figure S4 Sensitivity analysis for DBP
