# Supplementary material for: Association of cooking fuel type with hypertension risk: a systematic review and meta-analysis
Source: BMC Public Health. 2026 Jan 7;26:386. doi: 10.1186/s12889-025-26168-5 (PMC12853769; doi:10.1186/s12889-025-26168-5)
Supplement: Supplementary file 2 — Supplementary Material 2: Table S1 PRISM checklist. Table S2 Pubmed search strategy. Table S3 Embase search strategy. Table S4 Cochrane Library search strategy. Table S5 Web of Science search strategy. Table S6 CNKI search strategy. Table S7 VIP search strategy. Table S8 Wanfang search strategy. Table S9 SinoMed search strategy. Table S10 Quality assessment of case-control studies. Table S11 Quality assessment of cohort studies. Table S12 Quality assessment of cross-sectional studies. Table S13 Previous meta-analysis summary table. [file 12889_2025_26168_MOESM2_ESM.docx]

Table S1 PRISM Checklist

| **Section and Topic** | **Item #** | **Checklist item** | **Location where item is reported** |
| --- | --- | --- | --- |
| **TITLE** | | |  |
| Title | 1 | Identify the report as a systematic review. | 1 |
| **ABSTRACT** | | |  |
| Abstract | 2 | See the PRISMA 2020 for Abstracts checklist. | 2-3 |
| **INTRODUCTION** | | |  |
| Rationale | 3 | Describe the rationale for the review in the context of existing knowledge. | 3-5 |
| Objectives | 4 | Provide an explicit statement of the objective(s) or question(s) the review addresses. | 3-5 |
| **METHODS** | | |  |
| Eligibility criteria | 5 | Specify the inclusion and exclusion criteria for the review and how studies were grouped for the syntheses. | 6-7 |
| Information sources | 6 | Specify all databases, registers, websites, organisations, reference lists and other sources searched or consulted to identify studies. Specify the date when each source was last searched or consulted. | 5-6 |
| Search strategy | 7 | Present the full search strategies for all databases, registers and websites, including any filters and limits used. | 5-6, Supplementary Tables S2–S9 |
| Selection process | 8 | Specify the methods used to decide whether a study met the inclusion criteria of the review, including how many reviewers screened each record and each report retrieved, whether they worked independently, and if applicable, details of automation tools used in the process. | 7 |
| Data collection process | 9 | Specify the methods used to collect data from reports, including how many reviewers collected data from each report, whether they worked independently, any processes for obtaining or confirming data from study investigators, and if applicable, details of automation tools used in the process. | 8-9 |
| Data items | 10a | List and define all outcomes for which data were sought. Specify whether all results that were compatible with each outcome domain in each study were sought (e.g. for all measures, time points, analyses), and if not, the methods used to decide which results to collect. | 8-10 |
|  | 10b | List and define all other variables for which data were sought (e.g. participant and intervention characteristics, funding sources). Describe any assumptions made about any missing or unclear information. | 8-10 |
| Study risk of bias assessment | 11 | Specify the methods used to assess risk of bias in the included studies, including details of the tool(s) used, how many reviewers assessed each study and whether they worked independently, and if applicable, details of automation tools used in the process. | 9-10 |
| Effect measures | 12 | Specify for each outcome the effect measure(s) (e.g. risk ratio, mean difference) used in the synthesis or presentation of results. | 9-10 |
| Synthesis methods | 13a | Describe the processes used to decide which studies were eligible for each synthesis (e.g. tabulating the study intervention characteristics and comparing against the planned groups for each synthesis (item #5)). | 8-10 |
|  | 13b | Describe any methods required to prepare the data for presentation or synthesis, such as handling of missing summary statistics, or data conversions. | 8-10 |
|  | 13c | Describe any methods used to tabulate or visually display results of individual studies and syntheses. | 8-10 |
|  | 13d | Describe any methods used to synthesize results and provide a rationale for the choice(s). If meta-analysis was performed, describe the model(s), method(s) to identify the presence and extent of statistical heterogeneity, and software package(s) used. | 8-10 |
|  | 13e | Describe any methods used to explore possible causes of heterogeneity among study results (e.g. subgroup analysis, meta-regression). | 8-10 |
|  | 13f | Describe any sensitivity analyses conducted to assess robustness of the synthesized results. | 9-10 |
| Reporting bias assessment | 14 | Describe any methods used to assess risk of bias due to missing results in a synthesis (arising from reporting biases). | 9-10 |
| Certainty assessment | 15 | Describe any methods used to assess certainty (or confidence) in the body of evidence for an outcome. | 9-10 |
| **RESULTS** | | |  |
| Study selection | 16a | Describe the results of the search and selection process, from the number of records identified in the search to the number of studies included in the review, ideally using a flow diagram. | 10-11, Figure1 |
|  | 16b | Cite studies that might appear to meet the inclusion criteria, but which were excluded, and explain why they were excluded. | 10-11, Figure1 |
| Study characteristics | 17 | Cite each included study and present its characteristics. | 11-12 |
| Risk of bias in studies | 18 | Present assessments of risk of bias for each included study. | 12-13 |
| Results of individual studies | 19 | For all outcomes, present, for each study: (a) summary statistics for each group (where appropriate) and (b) an effect estimate and its precision (e.g. confidence/credible interval), ideally using structured tables or plots. | 11-12 |
| Results of syntheses | 20a | For each synthesis, briefly summarise the characteristics and risk of bias among contributing studies. | 12-14 |
|  | 20b | Present results of all statistical syntheses conducted. If meta-analysis was done, present for each the summary estimate and its precision (e.g. confidence/credible interval) and measures of statistical heterogeneity. If comparing groups, describe the direction of the effect. | 12-14 |
|  | 20c | Present results of all investigations of possible causes of heterogeneity among study results. | 12-14 |
|  | 20d | Present results of all sensitivity analyses conducted to assess the robustness of the synthesized results. | 14 |
| Reporting biases | 21 | Present assessments of risk of bias due to missing results (arising from reporting biases) for each synthesis assessed. | 14 |
| Certainty of evidence | 22 | Present assessments of certainty (or confidence) in the body of evidence for each outcome assessed. | 14 |
| **DISCUSSION** | | |  |
| Discussion | 23a | Provide a general interpretation of the results in the context of other evidence. | 15-19 |
|  | 23b | Discuss any limitations of the evidence included in the review. | 19-20 |
|  | 23c | Discuss any limitations of the review processes used. | 19-20 |
|  | 23d | Discuss implications of the results for practice, policy, and future research. | 20-21 |
| **OTHER INFORMATION** | | |  |
| Registration and protocol | 24a | Provide registration information for the review, including register name and registration number, or state that the review was not registered. | 5 |
|  | 24b | Indicate where the review protocol can be accessed, or state that a protocol was not prepared. | 5 |
|  | 24c | Describe and explain any amendments to information provided at registration or in the protocol. | 5 |
| Support | 25 | Describe sources of financial or non-financial support for the review, and the role of the funders or sponsors in the review. | 22 |
| Competing interests | 26 | Declare any competing interests of review authors. | 22 |
| Availability of data, code and other materials | 27 | Report which of the following are publicly available and where they can be found: template data collection forms; data extracted from included studies; data used for all analyses; analytic code; any other materials used in the review. | 23 |

*From:*  Page MJ, McKenzie JE, Bossuyt PM, Boutron I, Hoffmann TC, Mulrow CD, et al. The PRISMA 2020 statement: an updated guideline for reporting systematic reviews. BMJ 2021;372:n71. doi: 10.1136/bmj.n71

Table S2 Pubmed Search Strategy

| Pubmed | | |
| --- | --- | --- |
| # | Query | Results |
| 1 | "Hypertension"[MeSH Terms] | 326,781 |
| 2 | "Blood Pressure"[MeSH Terms] | 314,040 |
| 3 | "vascular pressure"[Title/Abstract] OR "Systolic Pressures"[Title/Abstract] OR "Systolic Pressure"[Title/Abstract] OR "systolic blood pressure"[Title/Abstract] OR "systemic hypertension"[Title/Abstract] OR "secondary hypertension"[Title/Abstract] OR "salt hypertension"[Title/Abstract] OR "salt high blood pressure"[Title/Abstract] OR "Pulse Pressure"[Title/Abstract] OR "preexistent hypertension"[Title/Abstract] OR "normotension"[Title/Abstract] OR "neurogenic hypertension"[Title/Abstract] OR "intravascular pressure"[Title/Abstract] OR "hypertensive response"[Title/Abstract] OR "hypertensive reaction"[Title/Abstract] OR "hypertensive effect"[Title/Abstract] OR "hypertensive disease"[Title/Abstract] OR "Hypertension"[Title/Abstract] OR "HTN "[Title/Abstract] OR "high renin hypertension"[Title/Abstract] OR "High Blood Pressures"[Title/Abstract] OR "High Blood Pressure"[Title/Abstract] OR "endocrine hypertension"[Title/Abstract] OR "Diastolic Pressure"[Title/Abstract] OR "diastolic blood pressure"[Title/Abstract] OR "controlled hypertension"[Title/Abstract] OR "cardiovascular hypertension"[Title/Abstract] OR "blood tension"[Title/Abstract] OR "blood systolic pressure"[Title/Abstract] OR "blood pressure, high"[Title/Abstract] OR "blood pressure"[Title/Abstract] OR "blood diastolic pressure"[Title/Abstract] OR "arterial hypertension"[Title/Abstract] OR "acute hypertension"[Title/Abstract] | 749,730 |
| 4 | "cooking"[MeSH Terms] | 15,245 |
| 5 | "cooking"[Title/Abstract] OR "cookery"[Title/Abstract] OR "cooking oil fumes"[Title/Abstract] OR "clean household fuels"[Title/Abstract] OR "solid fuel"[Title/Abstract] OR "clean fuel"[Title/Abstract] OR "cooking fuel"[Title/Abstract] OR "biomass fuel"[Title/Abstract] OR "cooking exposure"[Title/Abstract] | 23,429 |
| 6 | (#1 OR #2 OR #3) AND (#4 OR #5) | 574 |

Table S3 Embase Search Strategy

| Embase | | |
| --- | --- | --- |
| # | Query | Results |
| 1 | 'hypertension'/exp | 1085375 |
| 2 | 'systolic blood pressure'/exp | 228523 |
| 3 | 'diastolic blood pressure'/exp | 139693 |
| 4 | 'blood pressure'/exp | 773593 |
| 5 | 'vascular pressure':ti,ab,kw OR 'systolic pressures':ti,ab,kw OR 'systolic pressure':ti,ab,kw OR 'systolic blood pressure':ti,ab,kw OR 'systemic hypertension':ti,ab,kw OR 'secondary hypertension':ti,ab,kw OR 'salt hypertension':ti,ab,kw OR 'salt high blood pressure':ti,ab,kw OR 'pulse pressure':ti,ab,kw OR 'preexistent hypertension':ti,ab,kw OR 'normotension':ti,ab,kw OR 'neurogenic hypertension':ti,ab,kw OR 'intravascular pressure':ti,ab,kw OR 'hypertensive response':ti,ab,kw OR 'hypertensive reaction':ti,ab,kw OR 'hypertensive effect':ti,ab,kw OR 'hypertensive disease':ti,ab,kw OR 'hypertension':ti,ab,kw OR 'htn':ti,ab,kw OR 'high renin hypertension':ti,ab,kw OR 'high blood pressures':ti,ab,kw OR 'high blood pressure':ti,ab,kw OR 'endocrine hypertension':ti,ab,kw OR 'diastolic pressure':ti,ab,kw OR 'diastolic blood pressure':ti,ab,kw OR 'controlled hypertension':ti,ab,kw OR 'cardiovascular hypertension':ti,ab,kw OR 'blood tension':ti,ab,kw OR 'blood systolic pressure':ti,ab,kw OR 'blood pressure, high':ti,ab,kw OR 'blood pressure':ti,ab,kw OR 'blood diastolic pressure':ti,ab,kw OR 'arterial hypertension':ti,ab,kw OR 'acute hypertension':ti,ab,kw | 1158533 |
| 6 | 'cooking'/exp | 26439 |
| 7 | 'cooking':ti,ab,kw OR 'cookery':ti,ab,kw OR 'cooking oil fumes':ti,ab,kw OR 'clean household fuels':ti,ab,kw OR 'solid fuel':ti,ab,kw OR 'clean fuel':ti,ab,kw OR 'cooking fuel':ti,ab,kw OR 'biomass fuel':ti,ab,kw OR 'cooking exposure':ti,ab,kw | 26184 |
| 8 | (#1 OR #2 OR #3 OR #4 OR #5) AND (#6 OR #7) | 1156 |

Table S4 Cochrane Library Search Strategy

| Cochrane Library | | |
| --- | --- | --- |
| # | Query | Results |
| 1 | MeSH descriptor: [Hypertension] explode all trees | 25500 |
| 2 | MeSH descriptor: [Blood Pressure] explode all trees | 34892 |
| 3 | ('vascular pressure' OR 'Systolic Pressures' OR 'Systolic Pressure' OR 'systolic blood pressure' OR 'systemic hypertension' OR 'secondary hypertension' OR 'salt hypertension' OR 'salt high blood pressure' OR 'Pulse Pressure' OR 'preexistent hypertension' OR 'normotension' OR 'neurogenic hypertension' OR 'intravascular pressure' OR 'hypertensive response' OR 'hypertensive reaction' OR 'hypertensive effect' OR 'hypertensive disease' OR 'Hypertension' OR 'HTN ' OR 'high renin hypertension' OR 'High Blood Pressures' OR 'High Blood Pressure' OR 'endocrine hypertension' OR 'Diastolic Pressure' OR 'diastolic blood pressure' OR 'controlled hypertension' OR 'cardiovascular hypertension' OR 'blood tension' OR 'blood systolic pressure' OR 'blood pressure, high' OR 'blood pressure' OR 'blood diastolic pressure' OR 'arterial hypertension' OR 'acute hypertension'):ti,ab,kw | 182701 |
| 4 | MeSH descriptor: [Cooking] explode all trees | 522 |
| 5 | (‘cooking' OR 'cookery' OR 'cooking oil fumes' OR 'clean household fuels' OR 'solid fuel' OR 'clean fuel' OR 'cooking fuel' OR 'biomass fuel' OR 'cooking exposure'):ti,ab,kw | 1920 |
| 6 | (#1 OR #2 OR #3) AND (#4 OR #5) | 294 |

Table S5 Web of Science search strategy

| Web of Science | | |
| --- | --- | --- |
| # | Query | Results |
| 1 | TS=("cooking" OR "cookery" OR "cooking oil fumes" OR "clean household fuels" OR "solid fuel" OR "clean fuel" OR "cooking fuel" OR "biomass fuel" OR "cooking exposure") | 76151 |
| 2 | TS=("vascular pressure" OR "Systolic Pressures" OR "Systolic Pressure" OR "systolic blood pressure" OR "systemic hypertension" OR "secondary hypertension" OR "salt hypertension" OR "salt high blood pressure" OR "Pulse Pressure" OR "preexistent hypertension" OR "normotension" OR "neurogenic hypertension" OR "intravascular pressure" OR "hypertensive response" OR "hypertensive reaction" OR "hypertensive effect" OR "hypertensive disease" OR "Hypertension" OR "HTN " OR "high renin hypertension" OR "High Blood Pressures" OR "High Blood Pressure" OR "endocrine hypertension" OR "Diastolic Pressure" OR "diastolic blood pressure" OR "controlled hypertension" OR "cardiovascular hypertension" OR "blood tension" OR "blood systolic pressure" OR "blood pressure, high" OR "blood pressure" OR "blood diastolic pressure" OR "arterial hypertension" OR "acute hypertension") | 881805 |
| 3 | #1 AND #2 | 739 |

Table S6 CNKI Search Strategy

| CNKI | | |
| --- | --- | --- |
| Query |  | Results |
| 1 | (Excerpt: hypertension + systolic + diastolic + blood pressure (blurry)) AND (Excerpt: cooking dyes + household fuels + solid fuels + clean fuels + biofuels + coal + natural gas + fuel types (blurry)) | 555 |

Table S7 VIP Search Strategy

| VIP | | |
| --- | --- | --- |
| # | Query | Results |
| 1 | ((((Any Field=Hypertension OR Any Field=Systolic) OR Any Field=Diastolic) OR Any Field=Blood Pressure) AND (((((((Any Field=Cooking Fuels OR Any Field=Household Fuels) OR Any Field=Solid Fuels) OR Any Field=Clean Fuels) OR Any Field=Biofuels) OR Any Field=Coal) OR Any Field=Natural Gas) OR Any Field=Fuel ​​Type)) | 1001 |

Table S8 Wanfang Search Strategy

| Wanfang | | |
| --- | --- | --- |
| # | Query | Results |
| 1 | TOPIC:("hypertension" OR "systolic blood pressure" OR "diastolic blood pressure" OR "blood pressure") and TOPIC:("cooking fuels" OR "household fuels" OR "solid fuels" OR "clean fuels" OR "biofuels" OR "coal" OR "natural gas" OR "fuel types") | 764 |

Table S9 SinoMed Search Strategy

| SinoMed | | |
| --- | --- | --- |
| # | Query | Results |
| 1 | ( "hypertension"[common field:intelligent] OR "systolic pressure"[common field:intelligent] OR "diastolic pressure"[common field:intelligent] OR "blood pressure"[common field:intelligent]) | 1408341 |
| 2 | ( "cooking fuel"[common field:intelligent] OR "household fuel"[common field:intelligent] OR "solid fuel"[common field:intelligent] OR "clean fuel"[common field:intelligent] OR "biofuel"[common field:intelligent]) OR( "coal"[common field:intelligent] OR "natural gas"[common field:intelligent] OR "fuel type"[common field:intelligent]) | 86708 |
| 3 | #1AND#2 | 723 |

Table S10 Quality assessment of case-control studies

| First Author | Q1 | Q2 | Q3 | Q4 | Q5 | Q6 | Q7 | Q8 | Scores |
| --- | --- | --- | --- | --- | --- | --- | --- | --- | --- |
| Alexander | 1 | 1 | 1 | 1 | 2 | 1 | 1 | 0 | 8 |
| Mitra | 1 | 1 | 1 | 1 | 2 | 1 | 1 | 1 | 9 |
| Younger | 1 | 1 | 1 | 1 | 1 | 1 | 1 | 0 | 7 |

Note:

Q1.Is the Case Definition Adequate?

Q2.Representativeness of the Cases

Q3.Selection of Controls

Q4.Definition of Controls

Q5.Comparability of Cases and Controls on the Basis of the Design or Analysis

Q6.Ascertainment of Exposure

Q7.Same method of ascertainment for cases and controls

Q8.Non-Response rate

Table S11 Quality assessment of cohort studies

| First Author | Q1 | Q2 | Q3 | Q4 | Q5 | Q6 | Q7 | Q8 | Scores |
| --- | --- | --- | --- | --- | --- | --- | --- | --- | --- |
| Zhang | 1 | 1 | 1 | 1 | 2 | 1 | 1 | 1 | 9 |
| Bellows | 1 | 1 | 1 | 1 | 2 | 1 | 1 | 0 | 8 |
| Kanagasabai | 1 | 1 | 1 | 1 | 2 | 1 | 0 | 1 | 8 |
| Peng | 1 | 1 | 0 | 1 | 2 | 1 | 1 | 1 | 8 |
| Su | 1 | 1 | 1 | 1 | 2 | 1 | 1 | 0 | 8 |
| Zhong | 1 | 1 | 0 | 1 | 2 | 1 | 1 | 0 | 7 |
| Yu | 1 | 1 | 1 | 0 | 2 | 1 | 0 | 0 | 6 |
| Weber | 1 | 1 | 0 | 1 | 2 | 0 | 0 | 0 | 5 |

Note:

Q1.Representativeness of the Exposed Cohort

Q2.Selection of the Non-Exposed Cohort

Q3.Ascertainment of Exposure

Q4.Demonstration That Outcome of Interest Was Not Present at Start of Study

Q5.Comparability of Cohorts on the Basis of the Design or Analysis

Q6.Assessment of Outcome

Q7.Was Follow-Up Long Enough for Outcomes to Occur

Q8.Adequacy of Follow Up of Cohorts

Table S12 Quality assessment of cross-sectional studies

| First Author | Q1 | Q2 | Q3 | Q4 | Q5 | Q8 | Q9 | Q11 | Q12 | Q14 | Quality Rating |
| --- | --- | --- | --- | --- | --- | --- | --- | --- | --- | --- | --- |
| Khan | YES | YES | CD | YES | NR | YES | YES | YES | CD | YES | Fair |
| Abba | YES | YES | CD | YES | NR | YES | YES | YES | CD | YES | Fair |
| Arku | YES | YES | CD | YES | YES | YES | YES | YES | CD | YES | Good |
| Ayebeng | YES | YES | CD | YES | NR | YES | YES | YES | CD | YES | Fair |
| Dutta | YES | YES | CD | YES | NR | YES | YES | YES | CD | YES | Fair |
| Islam | YES | YES | CD | YES | NR | YES | YES | YES | CD | YES | Fair |
| Lin | YES | YES | CD | YES | NR | YES | YES | YES | CD | YES | Fair |
| Liu | YES | YES | YES | YES | NR | YES | YES | YES | CD | YES | Good |
| Neupane | YES | YES | CD | YES | YES | YES | YES | YES | CD | YES | Good |
| Ofori | YES | YES | CD | YES | NR | YES | YES | YES | YES | YES | Good |
| Painschab | YES | YES | CD | YES | NR | YES | YES | YES | CD | YES | Fair |
| Yang | YES | YES | CD | YES | NR | YES | YES | YES | CD | YES | Fair |
| He | YES | YES | CD | YES | NR | YES | YES | YES | CD | YES | Fair |
| Wylie | YES | YES | CD | YES | NR | NR | YES | YES | CD | YES | Poor |
| Tiwana | YES | YES | CD | YES | NR | YES | YES | YES | CD | YES | Fair |
| Tawiah | YES | YES | CD | YES | YES | YES | YES | YES | CD | YES | Good |
| Juntarawijit | YES | YES | YES | YES | YES | YES | YES | YES | CD | YES | Good |
| Gu | YES | YES | YES | YES | YES | YES | YES | YES | CD | YES | Good |

Note: CD, cannot determine; NR, not reported

Q1. Was the research question or objective in this paper clearly stated?

Q2. Was the study population clearly specified and defined?

Q3. Was the participation rate of eligible persons at least 50%?

Q4. Were all the subjects selected or recruited from the same or similar populations (including the same time period)? Were inclusion and exclusion criteria for being in the study prespecified and applied uniformly to all participants?

Q5. Was a sample size justification, power description, or variance and effect estimates provided?

Q8. For exposures that can vary in amount or level, did the study examine different levels of the exposure as related to the outcome (e.g., categories of exposure, or exposure measured as continuous variable)?

Q9. Were the exposure measures (independent variables) clearly defined, valid, reliable, and implemented consistently across all study participants?

Q11. Were the outcome measures (dependent variables) clearly defined, valid, reliable, and implemented consistently across all study participants?

Q12. Were the outcome assessors blinded to the exposure status of participants?

Q14. Were key potential confounding variables measured and adjusted statistically for their impact on the relationship between exposure(s) and outcome(s)?

| PMID | Author | Year | Research Topic | Study Subjects and Sample Size | Exposure Factors | Outcome Measures | Main Conclusions |
| --- | --- | --- | --- | --- | --- | --- | --- |
| PMID: 31935611 | Lanyu Li | 2020 | The impact of solid fuel use on indoor air quality and its association with increased risk of hypertension. | 101262adults | Solid fuels (coal or biomass) used for cooking and heating | hypertension risk | The use of solid fuels in households significantly increases the risk of hypertension (odds ratio OR=1.27, 95% CI=1.06-1.51) |
| PMID: 29107891 | Raphael E Arku | 2018 | The relationship between cooking with solid fuels and elevated blood pressure as well as the risk of hypertension. | 77,605 premenopausal women aged 15-49 | Cooking using solid fuels (coal or biomass) | Systolic blood pressure, diastolic blood pressure, hypertension | Cooking with solid fuels is associated with a 7% increased risk of hypertension (OR=1.07, 95% CI=0.99-1.16） |
| PMID: 33645874 | David T Dillon | 2021 | The impact of biomass/solid fuel combustion on women's blood pressure. Biomass/solid fuels | 93,724 women | biomass/solid fuels | Systolic blood pressure, diastolic blood pressure, hypertension, vascular function, and inflammatory markers | Exposure to biomass fuels has a significant relationship with elevated blood pressure, but the relationship with hypertension is unclear. |

Table S13 Previous Meta-Analysis Summary Table
